# Supplementary material for: Expression and Transcript Localization of star, sf-1, and dax-1 in the Early Brain of the Orange-Spotted Grouper Epinephelus coioides
Source: Int J Mol Sci. 2022 Feb 27;23(5):2614. doi: 10.3390/ijms23052614 (PMC8910455; doi:10.3390/ijms23052614)
Supplement: Supplementary file 1 [file ijms-23-02614-s001.zip › ijms-1575607-SI.pdf]

```
GR-star     (1)    --PATFKLCAGISYRHMRNMTGLRKNALVAIHHELNRLAGPGPSNWISQVHRRSSLLSSR
JSB-star    (1)    MLPATFKLCAGISYRHMRNMTGLRKNAMVAIHHELNRLSGPGPSNWISQVRRRSSLLSSR
JA-star     (1)    MLPATFKLCAGISYRHMRNMTGLRKNAMVAIHHELNRLAGPGPSNWISQVRRRSSLLSSR
GSB-star    (1)    MLPATFKLCAGISYRHMRNMTGLRKNAMVAIHHELNRLAGPSPSNWISQVRRRSSLLSSR
LMB-star    (1)    MLPATFKLCAGISYRHMRNMTGLRKNAMVAIHHELNRLAGPGPSNWINQVRRRSSLLSSR
AC-star     (1)    MLPTTFKLCAGISYRHMRNMTGLRKNAMVAIHHELNRLAGPGSSNWISQVRRRSSFLGAR
ZFH-star    (1)    MLPATFKLCAGISYRHMRNMTGLRKNAMIAIHHELNKLSGPGASTWINHIRRRSSLLSSP
                     *.***********************..*******.*:**.:*.**:...****.*...

GR-star    (59)    IEEEQRYSEEEMSYVKQGENALQKAINILGEQDGWTIETVAANGDKVLSKVLPDIGKVFK
JSB-star   (61)    IEEEKGYNEEEMSYVKQGEDALQKAISILSEQDGWTIETVAANGDKVLSKMLPDIGKVFK
JA-star    (61)    IKEEDGYSEEEMSYVKQGEDALQKAIGILSEQDGWTIETVAANGDKVLSKVLPDVGKVFK
GSB-star   (61)    IREVEGYSEAEMSYVKQGEDALQKAISILSEQDGWTIETVAANGDKVLSKTLPDIGKVFK
LMB-star   (61)    IEEEEGYSDEEMSYVKQGEDALQKAISILSDQDGWTTEIVAANGDKVLSKMLPDIGKVFK
AC-star    (61)    IEEEQGYSEQEMSYVKQGEDALQKAISILSEQDGWTLETVAANGDKVLSKMLPDIGKVFK
ZFH-star   (61)    IAEET-YSEADQCYVQQGQEALQKSISILEDQDGWQTEIESINGEKVMSKVLPGIGKVFK
                   * *.  *..:...**.**..****.*:**::****.:*:.:.**.**.** **.:*****

GR-star   (119)    LEVMLEQHPDTLYEELVGNMEQMGEWNPNVKQVKILQKIGQDTMVTHEVSAETPGNVVGP
JSB-star  (121)    LEVMLEQHPDNLYEELVGNMEQMGEWNPNVKKVKILQKIGQDTMVTHEVSAETPGNVVGP
JA-star   (121)    LEVMLEQHPENLYEELVGKMEQMGEWNPNVKQVKILQKIGQDTMVTHEVSAETPGNVVGP
GSB-star  (121)    LEVMLEQHPDNLYEELVGNMEQMGDWNPNVKQVKILQKIGQDTMVTHEVSAETPGNVVGP
LMB-star  (121)    LEVMLEQRPDNLYKELVGNMEQMGEWNPNVKQVKILQKIGQDTMVTHEVSAETPGNVVGP
AC-star   (121)    LEVMLEQHPDNLYEELVGNMEQMGEWNPNVKQVKILQKIGKETMVTHEVSAETPGNVVGP
ZFH-star  (121)    LEVTLEQQTGDLYDELVDNMEQMGEWNPNVKQVKILQKIGQETMITHEISAETPGNVVGP
                   ***.***:.::**.***.:*****.******.********.:**.***.***********

GR-star   (179)    RDLVSVRCAKRRGSTCFLAGMSTQHAKMPEQRGVVRAENGPTCIVMKPCAEDPNKTKFTW
JSB-star  (181)    RDFVSVRCAKRRGSTCFLAGMSTQHPKMPEQRGVIRAENGPTCIVMKPCAEDPNKTKFTW
JA-star   (181)    RDFVSVRCAKRRGSTCFLAGMSTQHPRMPEQRGVVRAENGPTCIVMKPCAEDPNKTKFTW
GSB-star  (181)    RDFVSVRCAKRRGSTCFLAGMSTQHPKMPEQRGVVRAENGPTCIVMKPCAEDPNKTKFTW
LMB-star  (181)    RDFVSVRCAKRRGSTCFLAGMSTQHPKMPEQRGVVRAENGPTCIVMKPCVEDPNKTKFTW
AC-star   (181)    RDFVSVRCAKRRGSTCFLAGMSTKHPTMPEQRGVVRAENGPTCIVLKPCAEDPNKTKFTW
ZFH-star  (181)    RDFVNVRHAKRRGSTCFLAGMSTQHPGMPEQKGFVRAENGPTCIVMRPSADDPNKTKFTW
                   **.*.**.***************.*.:****.*..**********..*...*********

GR-star   (239)    LLSIDLKGWIPKTIINKVLSQTQV----------------------
JSB-star  (241)    LLNIDLKGWIPKTIINKVLSQTQVDFANHLRQRMANNVSMEMAHAC
JA-star   (241)    LLSIDLKGWIPKTIIHKVLSQTQVDFANHLRQRMANNVSMEMAHAC
GSB-star  (241)    LLNIDLKGWIPKTIINRVLSQTQVDFANHLRQRMANNVSMEMAHAC
LMB-star  (241)    LLNIDLKGWIPKTIINKVLSQTQVDFANHLRQRMANNVSMEMAHAC
AC-star   (241)    LLNIDLKGWIPKTIINKVLFQTQVDFASHLRQRMAHNVFMEMAHAC
ZFH-star  (241)    LLSLDLKGWIPKTVINRVLSQTQVDFVNHLRDRMASGGGIDAAIAC
                   **:.*********:*.:**.****
```

**Figure S1.**

```
GR-Sf1     (1)    ------------------------------------------------------------
JF-Sf1     (1)    MLPKVETESLGLSRSYGEHRHMPRIMQAPQIKMMDYSYDEDLDEMCPVCGDKVSGYHYGL
ESB-Sf1    (1)    MLPKVETESLGLSRSYGEQGHMPRNMQAPQLKMMDYSYDEDLDEMCPVCGDKVSGYHYGL
LMB-Sf1    (1)    --------------------------------MMDYSYDEDLDEMCPVCGDKVSGYHYGL
BP-Sf1     (1)    MLPKVETESLGLARSYGEQGHMPRNMQAHQLKMMDYSYDEDLDEMCPVCGDKVSGYHYGL
OH-Sf1     (1)    --------------------------------MMDYSYDEDLDEMCPVCGDKVSGYHYGL
ZFH-Sf1    (1)    MLPKVESEYLGLARSHGEQGHMPGNMQAPQFKMMDYSYDEDLDEMCPVCGDKVSGYHYGL


GR-Sf1     (1)    ---ESCKGFFKRTVQNNKRYTCIENQSCQIDKTQRKRCPYCRFQKCLTVGMKLEAVRADR
JF-Sf1     (61)   LTCESCKGFFKRTVQNNKRYTCIENQSCQIDKTQRKRCPYCRFQKCLTVGMKLEAVRADR
ESB-Sf1    (61)   LTCESCKGFFKRTVQNNKRYTCIENQSCQIDKTQRKRCPYCRFQKCLTVGMKLEAVRADR
LMB-Sf1    (29)   LTCESCKGFFKRTVQNNKRYTCIENQSCQIDKTQRKRCPYCRFQKCLTVGMKLEAVRADR
BP-Sf1     (61)   LTCESCKGFFKRTVQNNKRYTCIENQSCQIDKTQRKRCPYCRFQKCLTVGMKLEAVRADR
OH-Sf1     (29)   LTCESCKGFFKRTVQNNKRYTCIENQSCQIDKTQRKRCPYCRFQKCLTVGMKLEAVRADR
ZFH-Sf1    (61)   LTCESCKGFFKRTVQNNKRYTCIENQSCQIDKTQRKRCPYCRFQKCLTVGMKLEAVRADR
                     *********************************************************

GR-Sf1     (58)   MRGGRNKFGPMYKRDRALKQQKKALIRANGLKIEAMSQVMQAVPTDLTISSAIQNIHSAA
JF-Sf1     (121)  MRGGRNKFGPMYKRDRALKQQKKALIRANGLKIEAMTQVMQAVPTDLTISSAIQNIHSAA
ESB-Sf1    (121)  MRGGRNKFGPMYKRDRALKQQKKALIRANGLKIEAMTQVMQAVPTDLTISSAIQNIHSAA
LMB-Sf1    (89)   MRGGRNKFGPMYKRDRALKQQKKALIRANGLKIEAMTQVMQAVPTDLTITSAIQNIHSAA
BP-Sf1     (121)  MRGGRNKFGPMYKRDRALKQQKKALIRANGLKIEAMTQVMQAVPTDLTISSAIQNIHSAA
OH-Sf1     (89)   MRGGRNKFGPMYKRDRALKQQKKALIRANGLKIEAMSQVMQAVPTDLTISSAIQNIHSAA
ZFH-Sf1    (121)  MRGGRNKFGPMYKRDRALKQQKKALIRANGLKLEAMTQVMQTVPADLTITSAIQNIHSAS
                  ********************************.***:****.**.****:*********.

GR-Sf1     (118)  SKGLPLSHHPGHHTG---HHHHHHHHATALPPTDYDRSPFVTSPVSMAMPPHAGSLQGYQ
JF-Sf1     (181)  SKGLPLSHHAGHHTG-HHHHHHHHHHATALPPTDYDRSPFVTSPVSMAMPPHAGSLQGYQ
ESB-Sf1    (181)  SKGLPLSHHAGHHTG-HHHHHHHHHHATALPPTDYDRSPFVTSPVSMAMPPHAGSLQGYQ
LMB-Sf1    (149)  SKGLPLSHHPGHHTG-HHHHHHHHHHATALPPTDYDRSPFVTSPVSMAMPPHAGSLQGYQ
BP-Sf1     (181)  SKGLPLSHHTGHHAG-HHHHHHHHHHATALPPTDYDRSPFVTSPVSMAMPPHAGSLQGYQ
OH-Sf1     (149)  SKGLPLSHHAGHHTGHHHHHHHHYHHATALPPTDYDRSPFVTSPVSMAMPPHAGCLQGYQ
ZFH-Sf1    (181)  KG-----LPLSHHHHHHHHHHHHHSSSSAGLPPADFDRSPFVTSPVSMAMPPHAGGLQGYQ
                  ..     .. .**..   *****:.....***.*.*******************:*****

GR-Sf1     (175)  AAYGHFQGTRTIKSEYP-------------------------------------------
JF-Sf1     (240)  AAYGHFQGTRTIKSEYPDPYTSSPESIMGYAYVDAYQSGSPPSFPHLIVELLKCEPDEPQ
ESB-Sf1    (240)  AAYGHFQGTRTIKSEYPDPYTSSPESIMGYAYVDAYQSGSPPSFPHLIVELLKCEPDEPQ
LMB-Sf1    (208)  AAYGHFQGTRTIKSEYPDPYTSSPESIMGYAYVDAYQSGSPPSFPHLIVELLKCEPDEPQ
BP-Sf1     (240)  AAYGHFQGTRTIKSEYPDPYTSSPESIMGYAYVDAYQSGSPPSFPHLIVELLKCEPDEPQ
OH-Sf1     (209)  AAYGHFQGTRTINFEYPDPYTSSPESIMGYAYVDAYQSGSPPSFPHLIVELLKCEPDEPQ
ZFH-Sf1    (236)  AYG--HFQSRTIKSEYPDPYTSSPESLMGYPYVEAYAGGSPPSFPHLVVELLKCEPDEPQ
                  *..   ....***..***

GR-Sf1     (191)  ------------------------------------------------------------
JF-Sf1     (300)  VQAKILAYLQQEQASRGKHEKLNTFGLMCKMADQTLFSIVEWARSSIFFRELKVDDQMKL
ESB-Sf1    (300)  VQAKILAYLQQEQASRGKHEKLNTFGLMCKMADQTLFSIVEWARSSIFFRELKVDDQMKL
LMB-Sf1    (268)  VQAKILAYLQQEQASRGKHEKLNTFGLMCKMADQTLFSIVEWARSSIFFRELKVDDQMKL
BP-Sf1     (300)  VQAKILAYLQQEQASRGKHEKLNTFGLMCKMADQTLFSIVEWARSSIFFRELKVDDQMKL
OH-Sf1     (269)  VQAKILAYLQQEQASRGKHEKLNTFGLMCKMADQTLFSIVEWARSSIFFRELKVDDQMKL
ZFH-Sf1    (294)  VQAKILAYLQQEQASRGKHEKLNTFGLMCKMADQTLFSIVEWARSSIFFRELKVDDQMKL


GR-Sf1     (191)  ------------------------------------------------------------
JF-Sf1     (360)  LQNCWSELLILDHVFRQVVHAKEGSILLVTGQQVDYAVIASQAGATLNNLLSHAQELVAK
ESB-Sf1    (360)  LQNCWSELLILDHVFRQVVHAKEGSILLVTGQQVDYAVIASQAGATLNNLLSHAQELVGK
LMB-Sf1    (328)  LQNCWSELLILDHVFRQVVHAKEGSILLVTGQQVDYAVIASQAGATLNNLLSHAQELVAK
BP-Sf1     (360)  LQNCWSELLILDHVFRQVVHAKEGSILLVTGQQVDYAVIASQAGATLNNLLSHAQELVAK
OH-Sf1     (329)  LQNCWSELLILDHVFRQVVHAKEGSILLVTGQQVDYAMIASQAGATLNNLLSHAQELVTR
ZFH-Sf1    (354)  LQNCWSELLILDHVFRQVMHAKEGSILLVTGQQVDYALIASQAGATLNNLLSHAQELVSK


GR-Sf1     (191)  ------------------------------------------------------------
JF-Sf1     (420)  LRSLQLDQREFVCLKFLVLFSLDVKNLENFHLVESVQEQVNAALLDYVMCNYPQQTDKFG
ESB-Sf1    (420)  LRSLQLDQREFVCLKFLVLFSLDVKNLENFHLVESVQEQVNAALLDYVMCNYPQQTDKFG
LMB-Sf1    (388)  LRSLQLDQREFVCLKFLVLFSLDVKNLENFHLVESVQEQVNAALLDYVMCNYPQQTDKFG
BP-Sf1     (420)  LRSLQLDQREFVCLKFLVLFSLDVKNLENFHLVESVQEQVNAALLDYVMCNYPQQTDKFG
OH-Sf1     (389)  LRSLQLDQREFVCLKFLVLFSLDVKNLENFHLVESVQEQVNAALLDYVMCNYPQQTDKFG
ZFH-Sf1    (414)  LRSLQLDQREFVCLKFLVLFSLDVKNLENFHLVESVQEQVNAALLDYVMCNYPQQTDKFG


GR-Sf1     (191)  --------------------------------------------
JF-Sf1     (480)  QLLLRLPEIRAISLQAEEYLYYKHLNGDVPCNNLLIEMLHAKRA
ESB-Sf1    (480)  QLLLRLPEIRAISLQAEEYLYYKHLNGDVPCNNLLIEMLHAKRA
LMB-Sf1    (448)  QLLLRLPEIRAISLQAEEYLYYKHLNGDVPCNNLLIEMLHAKRA
BP-Sf1     (480)  QLLLRLPEIRAISLQAEEYLYYKHLNGDVPCNNLLIEMLHA---
OH-Sf1     (449)  QLLLRLPEIRAISLQAEEYLYYKHLNGDVPCNNLLIEMLHAKRA
ZFH-Sf1    (474)  QLLLRLPEIRAISLQAEEYLYYKHLNGDVPCNNLLIEMLHAKRA
```

**Figure S2.**

```
GR-Dax1     (1)   ------------------------------------------------------------
BP-Dax1     (1)   MATLEGCRCR-----SNNSILYSILKSDSLATTEQQQ--------QQQHPQQQLQHLLHK
ESB-Dax1    (1)   MATLEGCRCRGASGRNNNSILYNILKSDSLATAEEQQQQQQQQQQQQQQHPQQQTLQQLF
SE-Dax1     (1)   MATLEGCRCRGASGRNNSSILYSILKSNSLGTAEEEQ--------HPHPQQQQQQTLQHF
SS-Dax1     (1)   MATLEGCRCRGASGRDNNSILYSILKSDSLASAEEQQQNPQQQQQQQQQTWQHLLHKTSS
ZFH-Dax1    (1)   -MAYFDSGCHCSSERRQNSILYSILKNDSQSAQLGNQ-----------------------

GR-Dax1     (1)   ---------------------------------PQVTCKAASAVLVKTLRFVKNVPCFRE
BP-Dax1    (48)   TPLPSTAAPASLQELRQQPCSCGSTRRRGVLRSPQVTCKAASAVLVKTLRFVKNVPCFRE
ESB-Dax1   (61)   HKTSSTAAPASLQELRQQTCSCGSTRRRGILRFPQVTCKAASAVLVKTLRFVKNVPCFRE
SE-Dax1    (53)   FHKTLSSTPASLQEFRQQAYSCGSTRRLGILRSPQVTCKAASAVLVKTLRFVKNVPCFRE
SS-Dax1    (61)   TATTTSLQEHRQQEHRQQACSCGSMRRRGVLRSPQVTCKAASAVLVKTLRFVKNVPCFRE
ZFH-Dax1   (37)   -------PRAPRLAVSMRACACGSKRK-VSLRSPQTTCKAASAVLVKTLKFVKNVPCFRE
                                                   **.*************.**********

GR-Dax1    (28)   LPEDDRLMLIRSGWAPLLVLGLAQDRVDFETTETVEPSMLQRILTGVPDRQSEVLAGQS-
BP-Dax1   (108)   LPEDDQLMLIRSCWAPLLVLGLAQDRVDFETTETVEPSMLQRILTGVPDRQSEALAGQSS
ESB-Dax1  (121)   LPEDDQLMLIRSGWAPLLVLGLAQDRVDFETTETVEPSMLQRILTGLPDRQSEVPAGQS-
SE-Dax1   (113)   LPEDDQLMLIRSGWAPLLVLGLAQDRVDFETTETVEPSMLQRILTGLPDRQSDGPAGQS-
SS-Dax1   (121)   LPEDDQLTLIRSGWAPLLVLGLAQDRVDFETAETVEPSMLQRILTGLPDRQSEVLSGQS-
ZFH-Dax1   (90)   LPADDQHTLVRSGWAPLLVLGMAQDRIDFETSETQEPSMLQRILTSGQDKQDNQSHNGG-
                  **.**:.:*.**.********:****:****:**.**********.:.*.*.:  :...

GR-Dax1    (87)   RGAAGVSVVDIEAIKAFLKKCWSVDISTKEYAYLKGAVLFNPDVEGLRCLHYIQSLRREA
BP-Dax1   (168)   RGAAGVSVVDIEAIKAFLKKCWSVDISTKEYAYLKGAVLFNPDVEGLRCLHYIQSLRREA
ESB-Dax1  (181)   RGAAGVSVVDIEAIKAFLKKCWSVDISTKEYAYLKGAVLFNPDVEGLRCLHYIQSLRREA
SE-Dax1   (172)   RVAAGVSVVEIEAIKAFLKKCWSIDISTKEYAYLKGAVLFNPDLEGLRCLHYIQSLRREA
SS-Dax1   (|180)  RGAVGVSVLDIEAIKAFLKKCWSVDISTKEYAYLKGAVLFNPDVEGLRCPHYIQSLRREA
ZFH-Dax1  (149)   -----VALTDVQGIKMFLRKCWGLDISTKEYAYLKGAILFNPDVAGLQCQHYIQALQSEA
                       *.......**.**.***.:*************:*****:.**:*:****:*:.**

GR-Dax1   (147)   HQTLNEHVR---------------------------------------------------
BP-Dax1   (227)   HQALNEHVRLIHREDTTRFAKLLIALSMLRAISPPVVAQLFFRPVIGAVNIEEVLMEMFY
ESB-Dax1  (240)   HQALNEHVRLIHREDTTRFAKLLIALSMLRAINPLVVAQLFFRPVIGAVNIEEVLMEMFY
SE-Dax1   (240)   HQALNEHVRLIHREDTTRFAKLLIALSMLRAISPPVVAQLFFRPVIGTVSIEEVLLEMFY
SS-Dax1   (232)   HQALNEHVRLIHREDTTRFAKLLIALSMLRAISPPVVAQLFFRPVIGTVNIDEVLMGMFY
ZFH-Dax1  (204)   NQALNEYVKMIHRGDSARFAKLFLALSMLRSINANVVAGLFFKPVIGAVNMEELLLEMFY
                  .*.***.*.

GR-Dax1   (155)   --
BP-Dax1   (287)   --
ESB-Dax1  (301)   GK
SE-Dax1   (292)   GK
SS-Dax1   (300)   GK
ZFH-Dax1  (263)   GK
```

**Figure S3.**

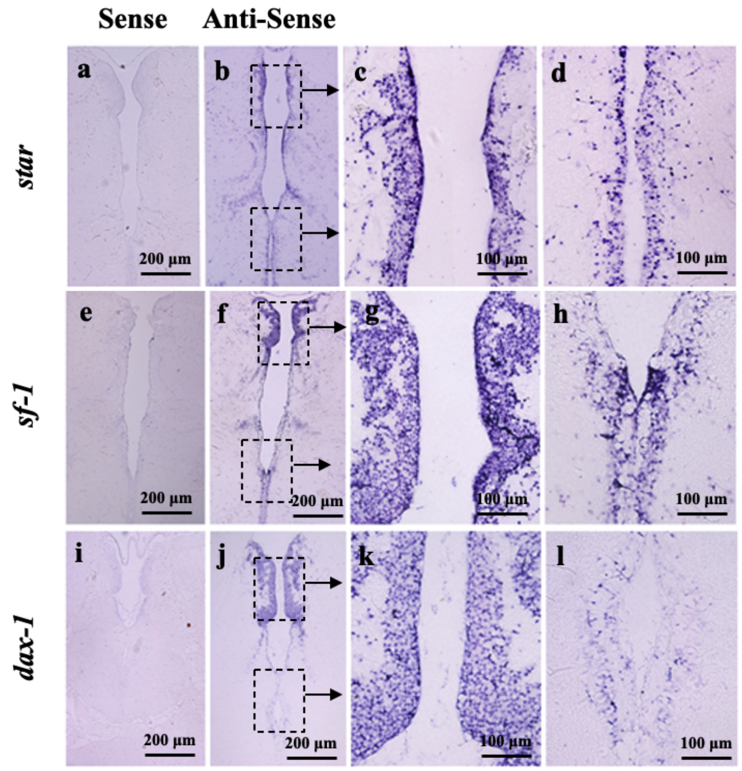


**Figure S4.**

Paint X lite

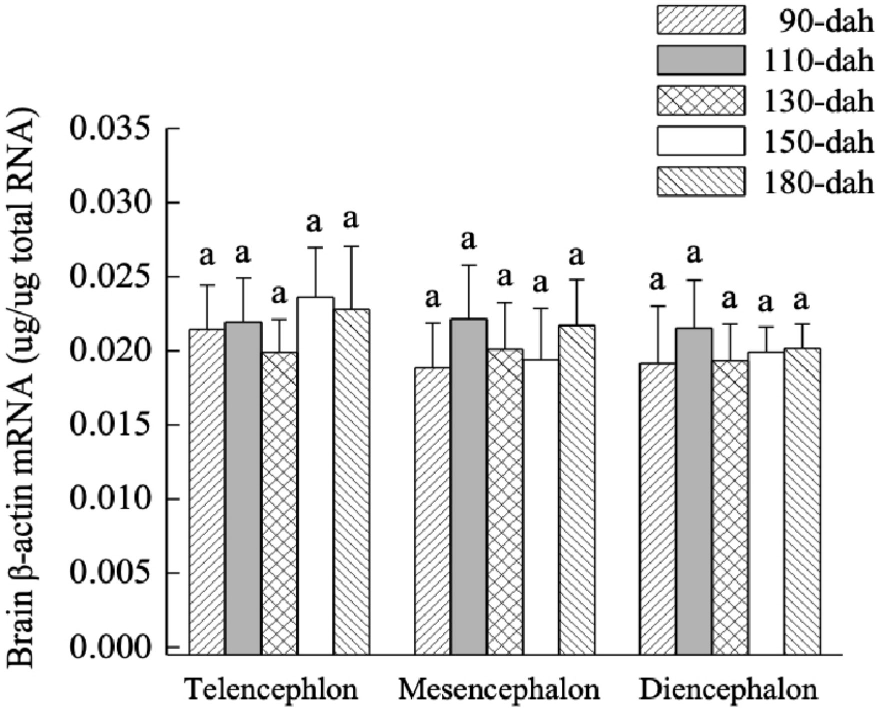


**Figure S5.**

Paint X lite
